# A systematic review of opioid prevalence in Australian residential aged care facilities

**DOI:** 10.1111/ajag.13071

**Published:** 2022-04-08

**Authors:** Laura A. Dowd, Lorenna Reynolds, Amanda J. Cross, Felicity Veal, Michelle Steeper, Zainab Wanas, Nancy Wu, J. Simon Bell

**Affiliations:** ^1^ Centre for Medicine Use and Safety Faculty of Pharmacy and Pharmaceutical Sciences Monash University Parkville, Melbourne Victoria Australia; ^2^ 3925 Unit for Medication Outcomes Research & Education (UMORE) School of Pharmacy and Pharmacology University of Tasmania Hobart Tasmania Australia; ^3^ National Health and Medical Research Council (NHMRC) Centre of Research Excellence in Frailty and Healthy Ageing Adelaide South Australia Australia

**Keywords:** aged, analgesics, opioid, pain, pain management

## Abstract

**Objective:**

To systematically review the prevalence of opioid prescribing, dispensing and administration in Australian residential aged care facilities (RACFs).

**Methods:**

MEDLINE, Embase, CINAHL, AgeLine, Web of Science Core Collection, InformIT and International Pharmaceutical Abstracts (inception to September 2021) were searched for studies reporting opioid prevalence in Australian RACFs. Regular and as‐required (i.e. pro re nata, PRN) opioid uses were considered. Screening, data extraction and quality assessment were performed independently by two review authors.

**Results:**

Twenty‐three studies (*n* = 286,141 residents) reported opioid prevalence, of which 16 provided overall regular or PRN prescribing, dispensing or administration data. Five studies reported 28%–34% of residents were prescribed regular opioids over assessment periods ranging from one week to one month. Five studies reported 11%–42% of residents were prescribed PRN opioids over assessment periods ranging from one week to 30 months. Three studies reported 27%–50% of residents were dispensed an opioid over 12 months. Five studies reported 21%–29% were administered both regular and PRN opioids over 24 hours. Two studies reported 22%–42% of residents were administered PRN opioids over 1 week to 12 months. Two studies reported 6%–13% of residents were using doses >100 mg oral morphine equivalents/day.

**Conclusions:**

Up to half of the residents were dispensed opioids over 12 months. The prevalence of opioid prescribing, dispensing and administration was highly variable, suggesting the potential value of opioid quality indicators and analgesic stewardship interventions to ensure opioid appropriateness.

**Practice Impact**

This systematic review identified highly variable opioid prescribing, dispensing and administration in Australian residential aged care facilities (RACFs), indicating that future quality improvement processes should prioritise opioid analgesic stewardship to minimise harm and maximise benefits.

## 1 INTRODUCTION

Untreated and unmanaged pain has been associated with reduced quality of life,^1^ changed behavioural symptoms^2^ and increased care dependency.^3^ When initiating pharmacological pain management, clinical practice guidelines recommend a multimodal, stepwise approach commencing with non‐opioid therapies, with the addition of opioids for moderate‐to‐severe pain when non‐opioid therapies alone are ineffective.^1^, ^4^, ^5^ Pain management in residential aged care facilities (RACFs) is important, with up to 20% of residents experiencing pain receiving no analgesics.^6^, ^7^, ^8^ Conversely, one study reported 97 of 153 residents (63%) with no self‐reported pain and 198 of 270 residents (73%) with no clinician‐observed pain were administered analgesics.^8^

Ensuring safe and effective opioid use is challenging because older adults are susceptible to adverse drug events (ADEs), including constipation, nausea, sedation, falls and fractures.^9^, ^10^ Opioids are one of three medication classes implicated in up to 60% of emergency department presentations for ADEs among older adults in the United States.^11^ The 2019 American Geriatrics Society Beers Criteria Update advise opioids be reserved for severe acute pain due to the risk of falls, fractures and sedative load (i.e. concomitant use of opioids and other central nervous system (CNS) medications).^12^ Age‐related decline in renal and hepatic function can lead to accumulation of metabolites and opioid toxicity.^13^, ^14^ For example, morphine has renally excreted active metabolites that can accumulate in mild renal impairment.^15^ Opioids without active metabolites or prolonged clearance, such as oxycodone, buprenorphine and fentanyl, may be preferable in frail older adults.^5^

No previous reviews have synthesised data on opioid prevalence in Australian RACFs. Knowledge of current opioid use can guide health‐care professionals, aged care providers and policymakers to address any potential under‐ and overuse. The objective was to systematically review the prevalence of opioid prescribing, dispensing and administration in Australian RACFs.

## 2 METHODS

A review protocol was prepared in advance with support from an experienced information specialist, but not registered. The remaining Preferred Reporting Items for Systematic Reviews and Meta‐Analyses (PRISMA) guidelines were followed.^16^

### 2.1 Eligibility

The review included original, peer‐reviewed articles. Participants were residents of Australian RACFs. Residential aged care facilities are synonymous with ‘nursing homes’ and ‘long‐term care facilities’ in other countries. In Australia, analgesics are predominantly prescribed by general medical practitioners (GPs), dispensed by off‐site community pharmacies and administered by qualified nurses.^17^ Included studies reported prescribing, dispensing or administration of overall regular or overall PRN opioid use and/or specific opioid medications. Studies utilising the same study sample were included where different characteristics of opioid use were reported. Specific opioids were defined as buprenorphine, codeine, fentanyl, hydromorphone, methadone, morphine, oxycodone, oxycodone and naloxone, pethidine, tapentadol and tramadol. There were no restrictions on study design. Studies that reported opioid initiation only were excluded. Studies that focused explicitly on cancer treatment and/or palliative care were excluded.

### 2.2 Search strategy

MEDLINE, Embase, CINAHL, AgeLine, Web of Science Core Collection, InformIT and International Pharmaceutical Abstracts were searched for studies published from inception to September 2021. Google, the Royal Commission into Aged Care Quality and Safety and Analysis & Policy Observatory, and reference lists of included studies were searched. The complete search strategy is shown in Appendix 1. When relevant conference abstracts were identified, attempts were made to identify potential full‐text articles.

### 2.3 Study selection and data extraction

The title and abstract screen, followed by a full‐text screen, were performed independently by two review authors (author 1 and author 2 or author 3). Any discrepancies were discussed until consensus was reached. A standardised data extraction tool was developed and pre‐piloted based on the ‘Joanna Briggs Institute (JBI) guidance for systematic reviews of prevalence and incidence studies’.^18^ If prevalence studies also reported elements of opioid appropriateness, this information was extracted. Criteria for assessing appropriateness were not prespecified but included analgesic co‐prescription, concomitant use of other CNS‐active drugs, ADEs and opioid dose.^5^, ^19^, ^20^ Data extraction was performed independently by a team of review contributors, then validated independently by two review authors (author 1 and author 2). Attempts were made to contact authors for additional information when needed.

### 2.4 Data synthesis

Results were synthesised for regular or PRN use, data source (i.e. prescribing, dispensing or administration records) and data collection period. For the purpose of the review, opioid prescribing was considered synonymous with charting. For studies reporting opioid prevalence based on Residential Medication Management Review (RMMR) records, this was considered under prescribing. Opioid use was calculated as the number of residents charted, dispensed or administered regular or PRN opioids as the numerator and the study sample as the denominator. Percentages were adjusted to whole numbers. For randomised controlled trials (RCTs), baseline data were used in the synthesis.

### 2.5 Quality assessment

Three review authors (author 1, author 2 and author 5) independently assessed the risk of bias using an adapted version of the ‘JBI Checklist for Prevalence Studies’ (Appendix 2, Table S3).^21^ The checklist was adapted to appraise the quality of opioid assessment in each study (i.e. not the overall quality of the study), irrespective of whether the studies were opioid‐related. Potential biases were assessed across seven criteria relating to sampling and opioid prevalence measurement. Sampling was considered appropriate if the study reported characteristics and demographics representative of the Australian RACF population. The measurement of opioid prevalence was considered to have a low risk of bias if the authors differentiated between regular and PRN use, clearly described how opioid use was measured (e.g. training or experience of those extracting medication data) and clearly reported the numerator and denominator.

## 3 RESULTS

**FIGURE 1:**
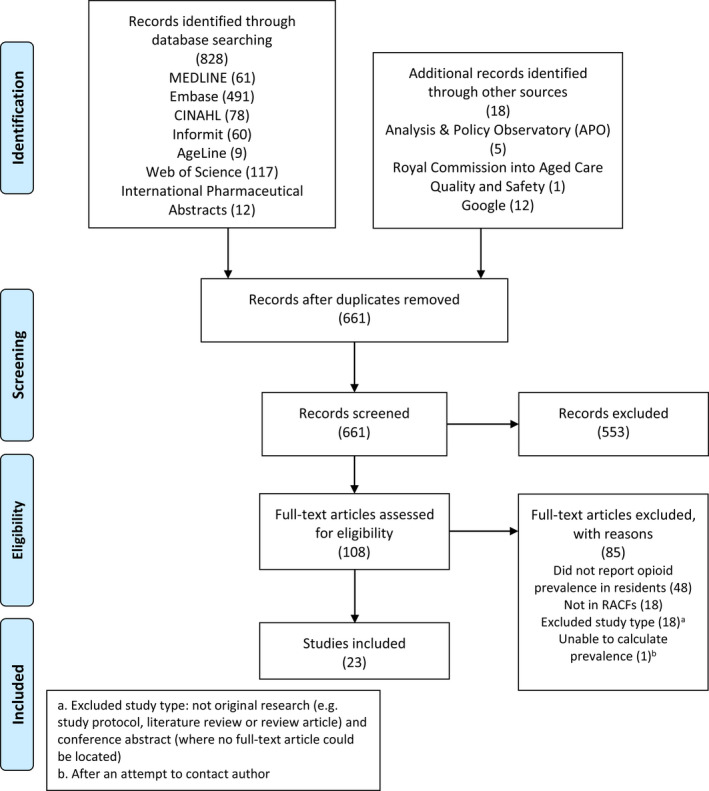
PRISMA flow chart of the study selection process

The search returned 846 articles, with 108 full‐text articles screened and 23 studies meeting the inclusion criteria and included in the final synthesis (Figure 1).

### 3.1 Characteristics

The 23 included studies involved 286,141 residents (Table 1, Table S1). The mean/median age of residents ranged from 81 to 91 years, and the proportion of female residents ranged from 58% to 78%. Study sample sizes ranged from 43 to 203,894.^22^, ^23^ Five^8^, ^24^, ^25^, ^26^, ^27^ and two studies^28^, ^29^ reported opioid prevalence utilising the same study sample. One RCT was included,^22^ and the remaining studies were observational. Of 22 observational studies, 18 reported the cross‐sectional prevalence of opioid use across various assessment periods.^8^, ^23^, ^24^, ^25^, ^26^, ^27^, ^28^, ^29^, ^30^, ^31^, ^32^, ^33^, ^34^, ^35^, ^36^, ^37^, ^38^, ^39^ One study combined a baseline cross‐sectional and 12‐month longitudinal audit.^40^ Three studies investigated period prevalence using a cohort design over 12 months,^41^ 24 months^42^ and 41 months.^43^ The majority of the included studies (18/23) were published in 2015 or later.^8^, ^22^, ^23^, ^24^, ^25^, ^26^, ^27^, ^29^, ^30^, ^31^, ^32^, ^34^, ^35^, ^38^, ^39^, ^40^, ^41^, ^43^ Data were sourced from South Australia,^8^, ^24^, ^25^, ^26^, ^27^ New South Wales,^33^, ^37^, ^42^ Victoria,^31^, ^40^ Queensland,^22^ Tasmania^39^ and Western Australia.^30^ Of 23 studies, 16 reported overall opioid prevalence (Table 1)^8^, ^22^, ^23^, ^24^, ^25^, ^27^, ^28^, ^30^, ^31^, ^33^, ^34^, ^37^, ^38^, ^39^, ^40^, ^41^ and 14 reported prevalence of specific opioids (‘*specific opioid classes’*, Table S2).^8^, ^24^, ^26^, ^27^, ^28^, ^29^, ^30^, ^32^, ^36^, ^39^, ^40^, ^41^, ^42^, ^43^ Definitions of opioid prevalence are provided in Table 1 and Table S1.

**TABLE 1:** Study characteristics and overall opioid prevalence

Author, year	Study design, study period	Setting, state	Opioid prevalence definition	Resident demographics		Overall opioid use/study sample (*n*/*N* (%))
				Mean age ± SD or median (IQR)	% female residents	
**Administered (*n* = 8)**						
Tan, 2015^27^ ^,^ ^a^ Tan, 2016^8^ ^,^ ^a^ Leung, 2015^24^ ^,^ ^a^	Cross‐sectional April–August 2014	6 RACFs, SA	Residents administered regular and/or PRN opioid(s) over 24 h	Mean 87.5 ± 6.2	78%	110/383 (29%)
Pont, 2018^34^	Cross‐sectional 1 October 2015	71 RACFs, NSW and ACT	Residents administered an opioid or opioid‐containing combination over 24 h	Mean 85.3 ± 8.5	71% (95% CI 69.3–71.9)	21.3% (95% CI 20.2–22.5)^b^ Opioids accounted for 26.0% (95% CI 24.8–27.3) of PRN administrations^c^
Hussein, 2019^30^	Cross‐sectional 1 May 2017	3 ACFs, WA	Residents administered an opioid over 24 h^d^	Mean 84 ± 8.5	64%	95/458 (21%)
Snowdon, 2006^37^ ^,^ ^e^	Cross‐sectional May–December 2003	50 RACFs, NSW	Residents administered regular opioid(s) over 2 weeks^d^	Mean 82.2	67%	Sample size: 4774 Regular morphine, codeine or other opioids (4%)^b^
Sharma, 2021^25^ ^,^ ^f^	Baseline cross‐sectional analysis of SIMPLER cluster RCT, 2017^61^	8 RACFs, SA	Opioids administered PRN in the previous 7 days for residents prescribed a PRN opioid at baseline	Median 87 (81–92)	74%	20 opioids administered of 90 residents prescribed PRN opioids at baseline (22%)
Picton, 2021^40^ ^,^ ^g^	Longitudinal audit, 1 July 2016–30 June 2017	10 RACFs, VIC	Residents administered PRN opioid(s) over 12 months^d^	Median 84 (76–90)	69%	PRN: 166/392 (42%)
**Prescribed (*n* = 10)**						
Jokanovic, 2017^31^	Cross‐sectional, May 2015	27 LTCFs, VIC	Residents prescribed regular opioid(s) as assessed on the audit date	Median 85.5 (78–90)	No polypharmacy: 68% Polypharmacy: 71%	258/754 (34%)
Picton, 2021^40^ ^,^ ^g^	Cross‐sectional, 1 July 2016^d^	10 RACFs, VIC	Residents prescribed PRN opioid(s) as assessed over 24 h^d^	Median 84 (76–90)	69%	118/392 (30%)
McClean, 2002^33^	Cross‐sectional, 1998–1999	15 NH, NSW	Residents self‐reporting pain and prescribed an opioid over seven days	Women 84.5 ± 8.7 Men 81.0 ± 4.5	72%	Sample size: 917 32% of those in present pain^b^
Veal, 2014^28^ ^,^ ^h^ Veal, 2015^29^ ^,^ ^h^	Retrospective cross‐sectional, January 2010–June 2012	RACFs, national	Residents prescribed regular and/or PRN opioid(s) as per RMMR record over the study period^d^	Mean 84.4 ± 9.1	69%	Regular 2057/7309 (28%) PRN 782/7309 (11%)
Veal, 2019^39^	Retrospective cross‐sectional, September–October 2015	5 ACFs, TAS	Residents prescribed regular and/or PRN opioid(s) as assessed over 24 h^d^	Median 85.1 (80.1–91.2)	65%	Regular 139/477 (29%) PRN 169/477 (35%)
Sharma, 2021^25^ ^,^ ^f^	Baseline cross‐sectional analysis of SIMPLER cluster RCT, 2017^61^	8 RACFs, SA	Residents prescribed regular and/or PRN opioid(s) over 24 h	Median 87 (81–92)	74%	Regular 73/242 (30%) PRN 90/242 (37%)
Pu, 2020^22^	Pilot RCT, January 2018–January 2019	3 LTCFs, state not specified	Residents prescribed regular and/or PRN opioid(s) over 7 days before intervention^d^	Mean 86 ± 7.4	70%	Regular 12/43 (28%) PRN 18/43 (42%)^d^, ^i^
Alderman, 2018^43^	Observational, 27 July 2013–1 January 2017	227 ACFs, five states	Opioid(s) prescribed as per RMMR report over the study period	N/A	N/A	N/A, specific classes only
Stasinopoulos, 2018^26^ ^,^ ^a^	Cross‐sectional, April–August 2014	6 RACFs, SA	Residents prescribed PRN and/or PRN + regular oxycodone over 7 days^d^	Median 88 (84–92)	78%	N/A, oxycodone only
**Dispensed (*n* = 7)**						
Kalisch Ellett, 2019^41^	Cohort, 1 July 2015–30 June 2016	ACFs, national	Veterans dispensed opioid(s) on the DVA administrative claims database during one year^d^	Median 91 (88–93)^j^	76%	7049/14,237 (50%)
Taxis, 2017^38^	Cross‐sectional, 2009	26 NHs, national	Residents with at least one dispensing of an opioid(s) over 1 year	Mean 85.8 ± 7.5	70%	Sample size 560 (43%)^b^, ^k^
Inacio, 2020^23^	Cross‐sectional, 2016	2690 ACFs, national	Long‐term residents dispensed an opioid for at least 90 days continuously, or for 120 non‐consecutive days over 1 year^d^	85.0 ± 8.8^d^	67%^d^	54,598/203,894 (26.8%, 95% CI: 26.6–26.9%)^k^, ^l^
Roughead, 2008^36^	Cross‐sectional, 1 April–30 June 2005	ACFs, national	Veterans with at least one dispensing of the opioid(s) in the 3‐month study period	Mean 86	58% (high care) 67% (low care)	N/A, specific classes only
Raban, 2020^35^	Cross‐sectional, 1 January 2017	66 RACFs, NSW and ACT	Residents supplied opioid‐containing patches by the pharmacy on the day of data extraction (24 h)	Mean 85.3 ± 8.0	69%	Opioid‐containing patches: 784/4784 (16%)
Dolton, 2012^42^	Cohort, July 2008–June 2010	26 ACFs, NSW	Residents who received one or more dispensings of tramadol during the two‐year study period^d^	Mean 82 ± 16^m^	67%^n^	N/A, tramadol only
Liu, 2019^32^	Cross‐sectional, January 2015–February 2016	17 NH, 4 states	Residents prescribed opioid(s) based on pharmacy records during the 1‐year study period^o^	Mean 85.5 ± 8.5	75%	N/A, buprenorphine, oxycodone and fentanyl only^k^

Abbreviations: ACFs, aged care facilities; DVA, Department of Veterans’ Affairs; LTCFs, long‐term care; NHs, nursing homes; PRN, Pro Re Nata; RACFs, residential aged care facilities; RCT, randomised controlled trial; RMMR, Residential Medication Management Review; SIMPLER, SImplification of Medications Prescribed to Long‐tErm care Residents.

^a^ Studies utilise data from the same study sample.

^b^ Reports percentage in manuscript only.

^c^ Use of occasional or ‘as‐needed’ medicines was 6.3% (95% CI: 5.7–7.0 of residents), and of these, opioids accounted for 26.0% (95% CI 24.8–27.3) of ‘as‐needed’ medicines.

^d^ As confirmed with the author.

^e^ Repeated cross section, took data from 2003 sample only (most recent), PRN medications given at least once daily for 4 weeks were considered regular. If a substantial number of the prescribed doses were not administered (even if prescribed to be given regularly), they were recorded as having been administered PRN (as confirmed with author).

^f^ Reported administration and prescribing data in the same study.

^g^ Reported point prevalence (prescribed) and period prevalence (administered) in the same study.

^h^ Utilised data from the same study sample. Veal et al.^29^ explored opioids in older Australians, including community‐based residents with a home medicine review record. Prevalence of specific opioids for RACF residents was reported separately in Veal et al.^29^

^i^ baseline prescribing only. Upon clarification with the author, 5 of 18 residents (28%) (1 in the intervention group and 4 in the control group) were administered a PRN opioid over the 6‐week intervention period.

^j^ At study entry.

^k^ Data obtained from supplementary information.

^l^ Short‐term residents were defined as having lived in a specific facility for a cumulative period of <100 days and long‐term residents for a cumulative period of ≥100 days. Chronic opioid use was defined as receiving any number of opioid medications for at least 90 days continuously or for 120 non‐consecutive days. The study did not examine intermittent or acute use. Facilities outside of the upper 95% CI chronic opioid use (= 277/2543, 11%).

^m^ Age information available for 3737 residents.

^n^ Sex information available for 3604 residents.

^o^ Prevalence = number of residents prescribed the opioid during the 1 year (regardless of frequency of use) as determined from the following sources: (1) pharmacy data records from nursing home‐contracted pharmacists; (2) facility‐based medication charts when pharmacy data could not be obtained; or (3) Pharmaceutical Benefits Scheme (PBS) data.

### 3.2 Prevalence of pain

One study reported that 28% of residents self‐reported pain, and of these residents reporting pain, 32% were prescribed opioids.^33^ Four studies reported the prevalence of pain and opioid use separately. In terms of clinician‐observed pain, one study reported 42% of residents were estimated to have moderate pain on the nurse‐estimated pain scale (0–10), with a mean score of 3.2 (±2.2).^22^ This study reported 28% of residents and 42% of residents were prescribed regular and/or PRN opioids at baseline, respectively. Veal et al.^39^ reported 26% of residents were observed to experience pain or reported pain during the preceding seven days. This study reported 29% of residents and 35% of residents were prescribed regular and PRN opioids at baseline, respectively. Two studies (utilising the same study sample) reported 30% of residents were observed to be in pain on the Pain Assessment in Advanced Dementia Scale (PAINAD), while 60% of residents self‐reported pain.^8^, ^27^ These studies reported 29% of residents were administered an opioid over a 24‐hour (24 h) period.

### 3.3 Prescribing

Five studies reported prescribing of regular opioids as per medical or RMMR records, ranging from 28% to 34% across various data collection periods. Two studies reported 29%^39^ and 30%^25^ of residents had a regular opioid charted over 24 h. Three studies reported 28% over 1 week,^22^ 28% over 30 months^28^ and 34% over 1 month had a regular opioid charted.^31^ No studies analysed GP prescribing records to determine opioid prevalence.

Five studies reported prescribing of PRN opioids, ranging from 11% to 42% across various data collection periods. Of 7309 residents indicated for a RMMR, 11% had a PRN opioid charted as recorded in the Medscope™ database.^28^ The dose and frequency of PRN opioid administration was not specified in the database. Three studies reported 30%,^40^ 35%^39^ and 37%^25^ of residents were charted a PRN opioid over a 24‐h data collection period. A pilot RCT reported 42% of 43 residents were charted a PRN opioid at baseline.^22^ One study did not specify whether opioids were charted for regular or PRN use. In this study, 32% were charted an opioid over a seven‐day data collection period.^33^

### 3.4 Dispensing

Three studies reported opioid prevalence based on pharmacy dispensing data, ranging from 27% to 50% over 12 months.^23^, ^38^, ^41^ It was not possible to determine whether dispensed opioids were intended for regular or PRN administration in these studies. The largest nationwide study (*n* = 203,894 long‐term residents) reported the prevalence of chronic opioid use was 27% in 2016. This study analysed Pharmaceutical Benefits Scheme (PBS) dispensing data from the Registry of Senior Australians (ROSA).^23^ Taxis et al.^38^ reported 43% of 1560 residents were dispensed an opioid in 2009. This study did not specify what type of database was used for data collection. The highest overall annual opioid prevalence was 50% among residents in the Australian Government Department of Veterans’ Affairs (DVA) dispensing claims database from July 2015 to June 2016.^41^

### 3.5 Administration

The lowest reported prevalence was 4% of 3054 residents administered regular opioids over two weeks from data collected in 2003.^37^ Five studies reported the administration of regular and PRN opioids over 24 h. Two of which extracted data from an electronic medication management system and reported 21% of residents were administered opioids.^30^, ^34^ Three studies that analysed administration data for 383 residents of six RACFs reported 29% were administered an opioid.^8^, ^24^, ^27^

Three studies reported the administration of PRN opioids and compared this with prescribing at baseline. In two studies, 37% of 242 residents and 30% of 392 were prescribed an opioid at baseline.^25^, ^40^ In these studies, 22% of residents were administered an opioid in the previous 7 days and 42% of residents were administered an opioid in the following 12 months. A pilot RCT investigating the effects of an interactive robotic companion seal (PARO) in people with dementia reported 42% of 43 residents were prescribed a PRN opioid at baseline. Over the 6‐week intervention, one resident in the intervention group and four residents in the control group were administered a PRN opioid.^22^ Three studies reported opioids were among the most frequently administered PRN medication class.^25^, ^34^, ^40^ One reported that opioids accounted for 26% of all PRN administrations.^34^

### 3.6 Opioid appropriateness

Of 23 studies reporting opioid prevalence, nine studies reported elements of opioid appropriateness.^24^, ^27^, ^28^, ^29^, ^30^, ^33^, ^41^, ^43^ Six studies reported analgesic co‐prescription.^27^, ^28^, ^29^, ^30^, ^33^, ^43^ Co‐administration of opioids and paracetamol ranged from 24%^27^ to 80%.^30^ One study reported 50% of residents charted regular opioids were co‐prescribed paracetamol at 3‐4 g/day and 48% were co‐prescribed anxiolytics/hypnotics.^28^ Kalisch Ellett et al.^41^ reported that the prolonged use of opioids was common, despite guideline recommendations for short‐term use. One study reported analgesic ADEs and found daytime sleepiness was not associated with opioid use in the previous 24 h.^27^

Three studies reported morphine milligram equivalents (MEQ)/day.^24^, ^28^, ^29^ One study reported the median MEQ/day was 21 mg, and 6% of residents administered opioids were administered doses exceeding the Australian maximum recommended dose of 100 mg MEQ/day for non‐cancer pain at the time of the study.^24^ Veal et al.^28^ reported the mean and median charted dose of regular opioids was 58 and 30 mg MEQ/day, respectively. In a second study by Veal et al.,^29^ 13% of residents charted opioids were charted doses exceeding 100 mg MEQ/day. Other studies reported analgesic load,^8^, ^27^ defined daily doses (DDD)^43^ and specific opioid doses.^30^ Of 23 studies, nine reported inabilities to assess or validate clinical indications, diagnoses or doses as a limitation.^8^, ^26^, ^27^, ^30^, ^31^, ^34^, ^36^, ^40^, ^41^

### 3.7 Specific opioid classes

Fourteen studies reported the prevalence of specific opioid classes (Table S2).^8^, ^24^, ^26^, ^27^, ^28^, ^29^, ^30^, ^32^, ^36^, ^39^, ^40^, ^41^, ^42^, ^43^ Oxycodone was the most prevalent opioid in seven studies and was dispensed in up to 28% of the study sample.^25^, ^26^, ^29^, ^30^, ^32^, ^41^, ^43^ Buprenorphine was the most prevalent opioid in four studies and was dispensed in up to 15% of the study sample.^8^, ^24^, ^27^, ^39^ The prevalence of opioids with active metabolites or which can accumulate in renal impairment (e.g. codeine, tramadol, morphine or hydromorphone) was <8% in all studies. The least prevalent opioids were methadone and pethidine.^43^

### 3.8 Quality assessment

Of 23 studies, three studies focused specifically on opioids,^24^, ^29^, ^30^ seven studies investigated pain, pain management or analgesics,^8^, ^22^, ^27^, ^28^, ^33^, ^39^, ^43^ while 13 studies reported opioid prevalence as part of a larger study.^23^, ^25^, ^26^, ^31^, ^32^, ^34^, ^35^, ^36^, ^37^, ^38^, ^40^, ^41^, ^42^ Sampling was considered appropriate in all but one study, which did not provide adequate information on how sampling was performed (Appendix 2, Table S3).^33^ Evidence of adequate sample sizes was provided in 11 studies.^8^, ^23^, ^24^, ^25^, ^26^, ^27^, ^28^, ^29^, ^36^, ^41^, ^43^ Reporting of PRN and/or regular opioid use was differentiated in 13 studies.^8^, ^22^, ^24^, ^25^, ^26^, ^27^, ^28^, ^29^, ^30^, ^31^, ^37^, ^39^, ^40^ Opioid administration to residents was reported in nine studies.^8^, ^22^, ^24^, ^25^, ^27^, ^30^, ^34^, ^37^, ^40^ Nine studies did not provide adequate information on how opioid prevalence was measured; therefore, reliability of methods could not be ascertained.^22^, ^32^, ^33^, ^34^, ^36^, ^38^, ^41^, ^42^, ^43^

## 4 DISCUSSION

This is the first systematic review to synthesise data on opioid prevalence in Australian RACFs. Twenty‐three studies reported opioid prevalence, of which 16 reported overall regular or PRN prescribing, dispensing or administration data and 14 reported the prevalence of specific opioids. The prevalence of opioid prescribing, dispensing and administration data varied widely across different assessment periods.

Kalisch Ellett et al.^41^ reported the highest overall opioid prevalence, with 1 in 2 residents dispensed an opioid over a 12‐month period. This study was conducted using the Australian Government DVA dispensing claims database. The DVA database included all reimbursed opioid dispensing for regular or PRN, short‐ or long‐term use, and end‐of‐life treatment. While dispensing records provide valuable data for pharmacoepidemiologic research, it was not possible to discern whether dispensed opioids were administered to residents.^44^ Conversely, studies reporting opioid administration tended to report lower prevalence overall, including 4% of residents administered regular opioids over 2 weeks in 2003.^37^ Following this, opioid rates began to increase across the board.^45^ This may be linked to greater awareness of untreated pain, clinicians’ desire to avoid non‐steroidal anti‐inflammatory drug (NSAID) adverse events, marketing of opioids and the addition of opioids to chronic non‐cancer pain guidelines. Several studies of opioid administration excluded residents with an estimated three months to live.^8^, ^24^, ^25^, ^26^, ^27^ This should be considered when interpreting prevalence as opioids are widely administered during end‐of‐life care.^40^ Studies reporting regular and/or PRN opioid administration provide arguably the most accurate reflection of opioid consumption at the resident level. Advances in information and communication technology (ICT) in RACFs can streamline the monitoring of medication administration and provide accurate resident‐level administration data.^46^

Three studies reported opioids were among the most administered PRN medications.^25^, ^34^, ^40^ In Australia, GPs visit RACFs periodically and PRN medications provide timely access to medications and ensure nursing staff have more scope to manage resident symptoms, such as breakthrough pain.^47^ One study reported PRN administration was more common among individuals residing in non‐metropolitan areas, where GP presence is more limited.^25^ The high rate of residents receiving both regular and PRN opioids raises the risk of exceeding maximum recommended daily doses,^48^ although this was found to be rare in two Australian studies that investigated this.^25^, ^26^ Three studies reported PRN opioid data assessed using more than one method.^22^, ^25^, ^40^ These studies showed differences in baseline prescribing and administration across various assessment periods, highlighting the importance of monitoring PRN opioid administrations in residents prescribed opioids. Evidence‐based guideline recommendations, regular assessments of pain and medication reviews can ensure the clinical appropriateness and safety of this practice.^49^, ^50^

It was encouraging that opioid selection was consistent with guideline recommendations to avoid opioids with the potential to accumulate in renal impairment.^4^, ^5^ Age‐related reduction in renal function or relative dehydration alters the elimination of morphine metabolites, particularly if taken regularly.^51^ Veal et al.^29^ reported that 13% of residents were charted doses higher than the recommended maximum for non‐cancer pain in Australian guidelines of 100 mg MEQ/day. The 2020 Royal Australian College of General Practitioners (RACGP) guideline recommends caution when increasing opioid dosage above 50 mg oral morphine equivalents (OME)/day and specialist involvement for doses greater than 100 mg OME/day.^5^ Changing guideline recommendations reflects an evolving understanding of opioid benefits and risks.

The high prevalence of opioid administration observed in Tan et al. (29% of residents administered an opioid over 24 h) was in the context of up to 60% of residents self‐reporting pain at the time of assessment.^8^, ^27^ Contextually, one in four adults aged 65 years and older experience chronic pain in Australia.^52^ In terms of opioid use internationally, there has been a moderate increase in regular and PRN opioid use in RACFs over the past 20 years, although geographical heterogeneity exists.^53^ There was an 8.5% decline in opioid prevalence in US nursing homes from 2011 to 2017, potentially reflecting a change in prescribing practices in response to the increased attention to the opioid epidemic in the United States.^54^ Further studies exploring international comparisons will allow for increased understanding of the cultural, medical and policy‐related driving forces on opioid use.

Defining opioid prevalence is important for understanding opioid burden in Australian RACFs. Pain management is broader than opioid use, and RACFs should have systems in place to improve, monitor and evaluate appropriate opioid prescribing and pain management.^55^ Nine studies in our review described the inability to determine opioid appropriateness as a limitation.^8^, ^26^, ^27^, ^30^, ^31^, ^34^, ^36^, ^40^, ^41^ It is recognised that the appropriate use of opioids requires an individualised assessment of pain and analgesic risks and benefits.^56^ Four studies reported pain and opioid use separately,^8^, ^22^, ^27^, ^39^ while one study reported the prevalence of opioid prescribing among residents self‐reporting pain.^33^ Multidisciplinary interventions that involve prescribers, nurses and pharmacists with a component of education have been demonstrated to improve analgesic use and appropriateness in RACFs.^57^

The prevalence of opioid prescribing, dispensing and administration was highly variable. This is consistent with the Australian Commission on Safety and Quality in Health Care identifying unexplained opioid variation in the general population.^58^ Potential reasons include differences in prescribing practices, lack of evidence‐based guideline recommendations and limited access to health‐care services, such as pain specialists and non‐pharmacological therapies, particularly in rural and remote locations. Although not yet specific to opioids, the National Aged Care Mandatory Quality Indicator Program initiated in 2019 provides the opportunity for benchmarking of medication prevalence across RACFs.^59^ Such systems provide a step towards efficiently monitoring variation and improving the quality use of medicines in RACFs. Further research exploring factors and outcomes associated with potential variation in opioid use across Australian RACFs has the potential to identify suboptimal practices and provide future targeted multidisciplinary analgesic stewardship interventions. In addition, the value of measuring opioid prevalence over consistent time frames would allow RACFs to review how opioid usage compares to other RACFs.

Strengths of this systematic review include the comprehensive synthesis of evidence across various assessment methods and definitions of opioid prevalence. Literature searching included grey literature and contacting nine authors. A broad range of studies conducted across most states of Australia were included. However, it is unclear to what extent the included studies represent all Australian RACFs. Limitations include a lack of meta‐analysis due to variability in study designs, data collection methods, length of data collection and reporting methods. This review does not include information on opioid‐naïve or incident users of opioids. Relatively, few studies have investigated opioid incidence in Australian RACFs. One study indicated that fentanyl patches were initiated in 2.6% of residents, and buprenorphine patches, in 8.7% over 3 months.^60^

## 5 CONCLUSIONS

Up to half of the residents of Australian RACFs were dispensed at least one opioid in a 12‐month period. The prevalence of opioid prescribing, dispensing and administration was highly variable, suggesting the potential value of opioid quality indicators and analgesic stewardship interventions to ensure opioid appropriateness.

## CONFLICTS OF INTEREST

No conflicts of interest declared.

## Supporting information

Supplementary Material

## Data Availability

The data that supports the findings of this study are available in the supplementary material of this article.
