# Supplementary material for: A systematic review of opioid prevalence in Australian residential aged care facilities
Source: Australas J Ageing. 2022 Apr 8;41(4):501–12. doi: 10.1111/ajag.13071 (PMC10083958; doi:10.1111/ajag.13071)
Supplement: Supplementary file 1 — Supplementary Material [file AJAG-41-501-s001.docx]

*Supplementary Information File*

**Appendix 1.** Search Strategy

MEDLINE (via Ovid)

exp Analgesics/ OR opioid*.mp. OR opiate*.mp. OR analgesi*.mp. OR morphine.mp. OR oxycodone.mp. OR codeine.mp. OR tapentadol.mp. OR fentanyl.mp. OR tramadol.mp. OR hydromorphone.mp. OR methadone.mp. OR pethidine.mp. OR buprenorphine.mp.

AND

exp Residential Facilities/ OR exp Health services for the aged/ OR exp Long-term care/ OR aged care.mp. OR residential facilit*.mp. OR nursing home*.mp. OR aged ADJ2 care.mp OR long term care.mp OR residential care.mp. OR age* ajd2 (home* or facilit*).mp.

AND

exp Australia/ OR Australia*.mp OR (New South Wales or NSW or Victoria* or VIC or South Australia* or SA or Western Australia* or WA or Northern Territory or NT or Queensland or QLD or Tasmania* or TAS or Australian Capital Territory or ACT).mp.

EMBASE (via Ovid)

exp opiate/ OR exp analgesic agent/ OR opioid*.mp. OR opiate*.mp. OR analgesi*.mp. OR morphine.mp. OR oxycodone.mp. OR codeine.mp. OR tapentadol.mp. OR tramadol.mp. OR fentanyl.mp. OR hydromorphone.mp. OR methadone.mp. OR pethidine.mp. OR buprenorphine.mp.

AND

exp nursing home/ OR exp elderly care/ OR exp nursing home patient/ OR exp residential care/ OR exp assisted living facility/ OR aged care.mp. OR residential facilit*.mp. OR nursing home*.mp. OR aged ADJ2 care.mp. OR long term care.mp. OR residential care.mp. OR age* adj2 (home* or facilit*).mp.

AND

exp Australia/ OR exp Australian/ OR australia*.mp. OR (New South Wales or NSW or Victoria* or VIC or South Australia* or SA or Western Australia* or WA or Northern Territory or NT or Queensland or QLD or Tasmania* or TAS or Australian Capital Territory or ACT).mp.

CINAHL (via EBSCOhost)

MH “Analgesics, Opioid+” OR MH “Narcotics+” OR opioid* OR opiate* OR analgesi* OR morphine OR oxycodone OR codeine OR tapentadol OR tramadol OR fentanyl OR hydromorphone OR methadone OR pethidine OR buprenorphine

AND

MH “Nursing Homes+” OR MH “Nursing Home Patients” OR MH “Gerontologic Care” OR MH “Age Specific Care” OR MH “Gerontologic Nursing+” OR MH “Long Term Care” OR MH “Housing for the Elderly” OR “residential care” OR “nursing home*” OR “aged care” OR (“long term care” or “long-term care”) OR “residential facilit*” OR “aged n2 care” OR age* n2 (home* or facilit*)

AND

MH “Australia+” OR Australia* OR (“New South Wales” or NSW or Victoria* or VIC or “South Australia*” or SA or “Western Australia*” or WA or “Northern Territory” or Queensland or QLD or Tasmania* or TAS or “Australian Capital Territory” or ACT)

*Note unable to search ‘NT’ in this database due to it being a field code.

AGELINE (via EBSCOhost)

DE “narcotics” OR opioid* OR opiate* OR analgesi* OR morphine OR oxycodone OR tapentadol OR buprenorphine OR methadone OR hydromorphone OR codeine OR fentanyl OR tramadol

AND

DE “For Profit Nursing Homes” OR DE “Homes for the Elderly” OR DE “Nonprofit Nursing Homes” OR DE “Nursing Homes” OR DE “Skilled Nursing Facilities” OR “nursing home*” OR “aged care” OR (“long term care” or “long-term care”)

AND

DE “Australia” OR DE “Australians” OR australia* OR (“New South Wales” or NSW or Victoria* or VIC or “South Australia*” or SA or “Western Australia*” or WA or “Northern Territory” or Queensland or QLD or Tasmania* or TAS or “Australian Capital Territory” or ACT)

Web of Science (Core Collection)

ALL= (Analgesi* OR opioid* OR opiate* OR codeine OR morphine OR oxycodone OR hydromorphone OR methadone OR pethidine OR tramadol OR tapentadol OR fentanyl OR buprenorphine)

AND

ALL= ("Aged care" OR "Residential care" OR "Nursing home*" OR "Long term care" OR "long-term care" OR "Residential facilit*")

AND

ALL= (australia* OR victoria* OR “new south wales” OR queensland OR “northern territory” “western australia*” OR “south australia” OR tasmania* OR “australian capital territory”)

Proquest - International Pharmaceutical Abstracts (IPA)

Analgesi* OR opioid* OR opiate* OR codeine OR morphine OR oxycodone OR hydromorphone OR methadone OR pethidine OR tramadol OR tapentadol OR fentanyl OR buprenorphine OR MAINSUBJECT.EXACT(“Analgesics and antipyretics”) OR MAINSUBJECT.EXACT(“Opiate antagonists”) OR MAINSUBJECT.EXACT(“Opiate agonists”) OR MAINSUBJECT.EXACT(“Opiate partial agonists”) OR MAINSUBJECT.EXACT(“Opiates”)

AND

“Aged care” OR “Residential care” OR “Nursing home*” OR “Long term care” OR “long-term care” OR “Residential facilit*” OR MAINSUBJECT.EXACT(“Nursing homes”) OR MAINSUBJECT.EXACT(“Long term care facilities”) OR MAINSUBJECT.EXACT(“Assisted living facilities”)

AND

australia* OR victoria* OR “new south wales” OR queensland OR “northern territory” “western australia*” OR “south australia” OR tasmania* OR “australian capital territory” OR MAINSUBJECT.EXACT(“Australia”)

INFORMIT

Analgesi* opioid* opiate* codeine morphine oxycodone hydromorphone methadone pethidine tramadol tapentadol fentanyl buprenorphine mh_phrase:Analgesics

AND

“Aged care” “Residential care” “Nursing home*” “Long term care” “long-term care” “Residential facilit*”

AND

australia* victoria* "new south wales" queensland "northern territory" "western australia*" "south australia" tasmania* "australian capital territory" mh_phrase:Australia

APO, Royal Commission into Aged Care Quality and Safety, Google

Using keywords including: opioid* or analgesi* AND aged care AND Australia

**Table S1**. Further characteristics of included studies

| **Author (Year)** | **Study Design** | **Inclusion/Exclusion criteria** | **Data collection methods** | **Setting,**  **State** | **Definition of opioid prevalence** | **Definition of aged care facility** | **Opioids  reported / any opioid specific exclusions** |
| --- | --- | --- | --- | --- | --- | --- | --- |
| Alderman 2018^43^ | Cohort | Inclusion: recipients of GP referred RMMR conducted between 27 July 2013 – 1 January 2017  Exclusion: duplicate reviews (i.e., subsequent  reviews for the same resident) | While performing medication reviews, details of medications prescribed (including dosage) were collated and stored in a secure database along with information about medical morbidities, demographic data and the results of clinical investigations. Details of analgesic and adjuvant medication used in pain management was subjected to de-identified analysis | 227 RACFs in 5 states of Australia (including NSW, VIC) | Number (%) of RMMR reports that had the opioid documented in the report (the pharmacist would have ‘documented all medications included in the active order documented on the medication chart’) | Unspecified. Included many RACF around Australia | Buprenorphine, codeine, fentanyl, hydromorphone, methadone, morphine, oxycodone, pethidine, tapentadol, tramadol, dextropropoxyphene In the RMMR report, the pharmacist should have documented the prescription, non-prescription, complementary/alternative medications included in the active order documented on the medication chart |
| Dolton 2012^42^ | Cohort | Inclusion: residents prescribed one or more medications from July 2008 – June 2010  Exclusion: not stated | Dispensing database | 26 long-term ACFs in Sydney, Australia | Number of residents dispensed tramadol out of the residents who were dispensed at least one drug | An aged-care facility was defined as including both low-level and high-level care, combining the traditional terminology of ‘nursing home’ or ‘aged care hostel’ | Tramadol. Interactions for medications not routinely used in the veteran population, not subsidised on the Australian Repatriation Pharmaceutical Benefits Scheme, and with a recommended dosage schedule less than 3 weeks, such as antibiotics, were excluded |
| Hussein 2019^30^ | Cross-sectional | N/A | Data was accessed using the iCare® platform and transcribed into a customized database. iCare® is a secure portal that comprises all resident details, medical history, observations, care plans, and medication administration, and is used for various quality assurance activities. Analgesic administration was the data collected. Study focused on ‘new’ administration of opioids. For oral therapy, the administration was the swallowing of a dose. Transdermal opioid patches that were replaced or affixed on the audit date were included, instead of an estimate of the proportion of the sustained-release dose received at the time of the audit | 3 ACFs in Perth, Australia | Number of residents who were administered the medication on the audit date (1 May 2017) | Audit using residents’ records from The Bethanie Group Inc., one of Western Australia’s aged care and retirement village providers. The data were sourced from three-aged care facilities, each ~160 beds and geographically diverse | Oxycodone/naloxone (Targin), oxycodone hydrochloride (Oxycontin, Oxynorm, Endone), buprenorphine (Norspan), tapentadol (Palexia), tramadol (Zydol), morphine hydrochloride (Ordine), paracetamol/codeine phosphate (CM strong pain relief, Panadeine), hydromorphone hydrochloride (Dilaudid, Jurnista), fentanyl (Durogesic) |
| Inacio 2020^23^ | Cross-sectional | Exclusion: Cancer (history of malignancy as reported at aged care eligibility assessment, entry into residential aged care assessment, and 6 months prior to reporting period history of antineoplastic agents) and palliative care (from entry into residential aged care assessment) | PBS | 2690 facilities across Australia | Number (%) of long-term residents that are chronic opioid users. Chronic opioid use is defined as receiving any number of opioid medications for at least 90 days continuously, or for 120 non-consecutive days. The number of days of medication use is determined based on the number of units dispensed and estimated dose per day. No gap days between one opioid medication dispensing and another were allowed when determining consecutive use of opioids | N/A | Morphine, hydromorphone, oxycodone, oxycodone + naloxone, fentanyl, methadone, methadone liquid, buprenorphine, codeine, combinations with paracetamol, tramadol, tapentadol |
| Jokanovic 2017^31^ | Cross-sectional | N/A | Researchers collected de-identified info from hardcopy or electronic medication charts and medical records | 27 LTCFs in regional and rural Victoria, Australia (all LTCFs from four health services) | Number (%) of patients with opioids regularly charted as per medication charts | LTCFs, synonymous with the term ‘nursing home’ or residential aged care facility, provides supported accommodation for people requiring functional support and nursing care | Unspecified. Once-only, telephone orders, nurse-initiated medications, as-needed and short-term medications excluded. Different formulations of the same medication were counted as separate medications; however, if multiple strengths were charted, the medication was counted only once. Combination products containing more than one active ingredient were coded as a single item |
| Kalisch Ellett 2019^41^ | Cohort | Inclusion: veterans eligible for all health services subsidised by DVA (‘gold card’ holders) and residing in continuous aged care at study start date  Exclusion: People receiving respite care, rehabilitation or in the community  and those who died prior to the end of the study period | DVA administrative claims data. The DVA administrative claims database contains details of all prescription medicines, medical, allied health services and hospitalisations provided to eligible Australian war veterans, their spouses and dependents | ACFs in Australia | Number (%) of residents dispensed an opioid over the 12-month period as per the DVA claim database | Unspecified care-need aged care facilities | No exclusions specified |
| Leung 2015^24^ | Letter to the editor displaying cross-sectional study (uses data from Tan et al.) | Inclusion: aged ≥ 65 years old, with or without dementia, able to participate in structured assessments in English  Exclusion: considered medically unstable, estimated life expectancy less than 3 months | Medication charts (described in another study using the same cohort) | 6 RACFs in South Australia (low level and high level). Located in metropolitan Adelaide and Mt Gambier | Number (%) of patients administered opioid in previous 24 hours as documented on the medication chart | Long-term care facilities | Fentanyl, oxycodone/naloxone, buprenorphine, tramadol, paracetamol+codeine, morphine, oxycodone. Includes prescription and non-prescription, both regular and PRN medications. Different formulations of the same active ingredient were considered different medications |
| Liu 2019^32^ | Cross-sectional | Inclusion: permanent residents of the facility, residing in the facility ≥ 12 months, not in immediate palliative care, if not able to self-consent - have family members able to provide proxy consent and/or participate on the resident’s behalf, no other complex medical or family issues that would prevent participation.  Exclusion: not stated | Pharmacy data records, facility-based medication charts when pharmacy data could not be obtained, or PBS data. When available, pharmacy records were used as the primary data source and then facility-based medication charts; where these were not available, PBS data were used. For each resident in the study, information regarding medication prescriptions in the previous 12 months was collected, including prescription and supply date. The number of different medications prescribed over the 12-month period was determined, regardless of frequency of use | 17 not-for-profit nursing homes across 4 Australian states | Number of residents prescribed the opioid during the 1-year period (regardless of frequency of use) as determined from the following sources: 1) pharmacy data records from nursing home contracted pharmacists; (2) facility-based medication charts when pharmacy data could not be obtained or (3) PBS data | Not-for-profit nursing homes | Oxycodone, fentanyl, buprenorphine, codeine (used for cough + analgesic) No specific exclusions stated. Prescription and non-prescription medications included |
| McClean 2002^33^ | Cross-sectional | N/A | Audit of medical records and interviews with residents. Medication data obtained from medical records, presence of pain etc obtained from patient interviews | 15 NH in northern NSW area health service (some charity-owned, some private for-profit) | % of patients (in pain and who were able to communicate) that had been prescribed opioid | Nursing home, some charity-owned, some private for-profit, rural | Unspecified. No specific opioid exclusions stated |
| Picton 2021^40^ | Baseline cross-sectional analysis and a longitudinal audit | Inclusion: all residents of the facilities | Data on all medications prescribed for scheduled or PRN administration on July 1, 2016 were extracted from each resident’s electronic medical record or hardcopy medication chart and recorded on the data collection form. Electronic medication administration data were included in the electronic medical record. Data on all administrations of pro re nata and nurse-initiated medicines for each resident for the follow-up period July 1, 2016 to June 30, 2017, or until the resident’s date of death or leaving the RACS were extracted | 10 RACFs in regional Victoria, Australia (Ballarat) [public sector] | Residents prescribed PRN opioid(s) as assessed on the index date and residents administered PRN opioid(s) during the 12-month period | Public-sector RACFS | Non stated. Included paracetamol and codeine combination products when assessed baseline prescribing only |
| Pont 2018^34^ | Cross-sectional | Inclusion: permanent residents in a participating RACF on 1 October 2015  Exclusion: short-stay respite care residents | De-identified demographic and medicine use data for permanent residents living in NSW or ACT Uniting facility on 1 October 2015 were extracted from the relevant clinical information systems. All Uniting facilities use an electronic clinical information system (iCareHealth) that includes an electronic medication administration record module | 71 RACFs in New South Wales and the Australian Capital Territory, Australia | Percentage of patients who were administered the medication in last 24 hours (as of 1st Oct 2015) | Uniting RACF | Unspecified - opioid or opioid containing combination No specific opioid exclusions stated |
| Pu 2020^22^ | Pilot RCT investigating the effects an interactive robotic companion seal (PARO) in people with dementia | Inclusion**:** (1) Age 65 years and older; (2) been diagnosed with some form of dementia, or probable diagnosis of dementia; and (3) Participants are assumed to experience underlying chronic pain (e.g., prescribed with regular pain medications or with an indication of pain); (4) Demonstration of perceived senses for interaction with PARO; and (5) Living in a facility for more than 3 months.  Exclusion: At least 1 of the following criteria: (1) Acute diseases (e.g., acute exacerbation of COPD) that required patients to be admitted to hospital frequently; (2) Terminal illnesses where the patient is in the final palliative stage; (3) A diagnosis of a major mental illness such as schizophrenia; (4) Infectious diseases such as acquired immune deficiency syndrome, or tuberculosis, or an open wound that was unable to be covered | Weekly recorded pain-related medications ordered on a regular basis and administered as needed and quantified by the Medication Quantification Scale-III.  As per author contact: the prescribing data at baseline and week 6 were collected. Medication consumption (administered) during the six-week intervention was also collected | 3 LTCFs, Australia | Number (%) of residents prescribed regular and PRN opioids in the week prior to the intervention (baseline) | N/A | Unspecified. No specific opioid exclusions stated |
| Raban 2020^35^ | Retrospective cross-sectional | Inclusion: permanent residents aged 65 years and over  Exclusion: no specific exclusions stated. Residents from two facilities with missing number of staff per bed data were excluded from the models | iCareHealth module. At the time of data extraction, transdermal patches were entered by the pharmacy in the same way as other medications. Facilities requested that pharmacies enter “check patch” and “remove patch” task reminders, so that they would appear during a medication round. The medication profiles contained all the medication data entered by pharmacies. The medication data consist of a medication name field and instructions field, both of which are free text. Residents who were using a transdermal patch at the time of data extraction were identified through a combination of text searches and medication codes, based on the transdermal patch preparations available locally. Those residents on patches containing opioid analgesics were flagged | 66 RACFs in New South Wales and the  Australian Capital Territory, Australia | Percentage of residents on patches, with their patch containing opioids | Residents in RACFs in Australia are treated and prescribed  medications by general practitioners and nurse practitioners  based in the community who visit the facilities | Not specified |
| Roughead 2008^36^ | Observational | Inclusion: veterans residing continuously in RACFs between 1 April – 30 June 2005, funded by DVA and with an active gold entitlement card  Exclusion: veterans with white and orange entitlement cards | DVA pharmacy claims database | ACFs in Australia | The proportion of veterans receiving medication dispensings between 1 April and 30 June 2005 (at least one dispensing during this time) | High and low-level care residential aged care facilities | Paracetamol with codeine, tramadol, oxycodone, other opioids, propoxyphene No opioid specific exclusions - but all medicines identified in the criteria requiring diagnostic information were excluded, as were all medicines not available on either the PBS or RPBS. |
| Sharma 2021^25^ | Secondary analyses of baseline and 12-month follow-up data from the SIMPLER cluster RCT (n=242 residents, 8 RACFs) | Inclusion: English-speaking permanent residents who took ≥1 medication regularly were recruited from 8 South Australian RACFs in 2017  Exclusion: residents anticipated to live for <3 months were excluded from participating at baseline | Details for all prescription and non-prescription medications (e.g., multivitamins, complementary and alternative medications) were extracted from paper-based medication charts and coded using the World Health Organization Anatomical Therapeutic Chemical (ATC) Classification System. Nurse-initiated medications, telephone orders, medications prescribed on the short-term section (e.g., antibiotics) and non-medications (e.g., bandages) were excluded | 8 RACFs, SA | Opioids administered PRN in the previous 7 days for residents prescribed a PRN opioid at baseline | Similar to long-term care facilities, RACFs provide accommodation and personal care for individuals who can no longer stay at home | Opioids (N02A) |
| Snowdon 2006^37^ | Cross-sectional | Data extracted for all residents in the RACFs at the time of the survey who had medication profiles available Inclusion/exclusion criteria not stated | Data were recorded from the medication cards and clinical files of all residents and were compared with data recorded in 1993 and 1998. Data collected by a research nurse. Confirmed with author: author checked whether medications had been given as prescribed during the previous 2 weeks, and if the prescription was PRN, the frequency of use in the previous 4 weeks was noted. If it was given at least once daily for 4 weeks, then it was regarded as a ‘regular’ prescription | 51 nursing homes in the Central Sydney Health Area | Residents administered regular opioid(s) over two-weeks | Nursing homes in the catchment area of Central Sydney Area Health Service | Morphine; codeine; other opioids (presented as one combined percentage)  No exclusions stated |
| Stasinopoulos 2018^26^ | A secondary analysis of cross-sectional data collected | Inclusion: aged ≥ 65 years old, with or without dementia, able to participate in structured assessments in English  Exclusion: considered medically unstable, estimated life expectancy less than 3 months | Details of medications (e.g., medication strength, directions for use) charted on a regular or PRN basis and number of doses administered over the preceding 7 days were extracted from medication charts | 6 RACFs in South Australia (low level and high level). Located in metropolitan Adelaide and Mt Gambier | Prevalence of charting (prescribing) and administration as per the medication charts. The number of patients with the medication charted, and the number of patients with the medication administered presented separately | Residential aged care services synonymous with ‘nursing homes’ or long-term care facilities, and provide assisted accommodation for people with care needs that can no longer be met at home | Oxycodone |
| Tan 2015^27^ | Cross-sectional | Inclusion: aged ≥ 65 years old, with or without dementia, able to participate in structured assessments in English  Exclusion: considered medically unstable, estimated life expectancy less than 3 months | Data was collected by three experienced study nurses. Demographic, diagnostic and medication data were extracted from each resident’s electronic medical record and medication chart. Other clinical data were collected using a standard data extraction form comprising a series of validated scales. Data on analgesic use in the previous 24 hours extracted from each resident’s medication chart | 6 RACFs, South Australia (metropolitan Adelaide and Mt Gambier) | Number (%) of patients administered opioid in previous 24 hours as documented on the medication chart | Low-level and high-level RACFs. In Australia, RACFs predominantly cater to older people who can no longer live at home due to frailty, disability or illness. RACFs may be funded through government, private or other organisations, and provide varying levels of support, ranging from daily tasks and personal care to 24-h care | Buprenorphine; fentanyl; oxycodone; oxycodone/naloxone All medications were extracted from the chart including prescription, non-prescription, complementary and alternative medications. Different formulations of the same active ingredient were considered different medications. Different strengths of the same active ingredient were considered the same medication. Includes prescription and non-prescription, both regular and PRN medications. Different formulations of the same active ingredient were considered different medications |
| Tan 2016^8^ | Cross-sectional | Inclusion: aged ≥ 65 years old, with or without dementia, able to participate in structured assessments in English  Exclusion: considered medically unstable, estimated life expectancy less than 3 months | Data was collected by three experienced study nurses. Demographic, diagnostic and medication data were extracted from each resident’s electronic medical record and medication chart. Other clinical data were collected using a standard data extraction form comprising a series of validated scales. Data on analgesic use in the previous 24 hours extracted from each resident’s medication chart | 6 RACFs in South Australia (low level and high level). Located in metropolitan Adelaide and Mt Gambier | Number (%) of patients administered opioid in previous 24 hours as documented on the medication chart | Low-level and high-level RACFs. In Australia, RACFs predominantly cater to older people who can no longer live at home due to frailty, disability or illness. RACFs may be funded through government, private or other organizations, and provide varying levels of support, ranging from daily tasks and personal care to 24-h care | Codeine; morphine; oxycodone; oxycodone/naloxone; fentanyl; buprenorphine; tramadol. Preparations containing combinations of paracetamol with codeine or dihydrocodeine were classified as paracetamol (N02BE51) if the codeine or dihydrocodeine content per unit dose was <20 mg. Otherwise, these combinations were classified as an opioid. Includes prescription and non-prescription, both regular and PRN medications. Different formulations of the same active ingredient were considered different medications |
| Taxis 2017^38^ | Cross-sectional | Inclusion: received one or more medications in 2009 | Conducted a cross-sectional analysis using pharmacy dispensing data, of all medications (prescription and non-prescription medications) used in 2009 by residents in 26 Australian and 6 Dutch nursing homes | 26 ACFs in Australia; 6 ACFs in Netherlands | Pharmacy dispensing data. Prevalence of medication use was defined as the number of individuals who received at least one supply of the medication during 2009 | Australian and Dutch nursing homes | Unspecified |
| Veal 2014^28^ | Retrospective | Inclusion: recipients of general practitioner referred RMMRs recorded in the Medscope database between January 2010 - June 2012.  Exclusion: RMMR reports that had no documented medical history were removed from analysis | Data from RMMRs recorded in Medscope database (recorded between Jan 2010-June 2012) | ACFs in Australia | Prevalence - % of residents prescribed opioid as per their RMMR report. Opioids classified as regular or PRN separately | Both high dependency (nursing home) and low dependency (residential home) care | Oxycodone (IR, CR), buprenorphine (patch), codeine ± combination, fentanyl (patch), morphine (IR, CR, injection), tramadol, other opioids used mainly as antitussives, or with directions indicating it is being used for cough or diarrhoea (dihydrocodeine, pholcodine, codeine linctus) were excluded |
| Veal 2015^29^ | Retrospective cross-sectional | Inclusion: recipients of general practitioner referred HMR and RMMRs recorded in the Medscope database between January 2010 - June 2012.  Exclusion: reports that had no documented medical history were removed from analysis | Reviewing patient records and medication reviews from Medscope | Community or ACFs in Australia | Prevalence = % of residents prescribed opioid | Unspecified | Buprenorphine (patch), oxycodone (IR, CR), fentanyl (patch), morphine (IR, CR), tramadol, codeine containing products. Opioids that were prescribed for non-pain indications were excluded from the analysis |
| Veal 2019^39^ | Retrospective review | Inclusion: permanent residents of the included RACFs  Exclusion: short-term respite residents. Some residents also excluded because they managed their medications themselves, medication charts were not available, or they died in the preceding week | Data extracted included results of the most recent assessment of pain and its management, frequency and treatment of pain incidents in the previous 7 days, demographics, and medical and medication history. Medical records used to obtain this information | 5 RACFs, Tasmania | Prevalence = number (%) of patients prescribed an opioid on the medication chart in the preceding 7 days | All ACFs in southern Tasmania, Australia with dementia and non-dementia specific beds were contacted to participate | Buprenorphine; oxycodone/naloxone; tramadol; paracetamol with codeine; codeine phosphate; oxycodone; morphine; fentanyl; hydromorphone; tapentadol; hydrocodone No exclusions of opioids reported |

Abbreviations; **ACFs**, Aged Care Facilities; **COPD**, Chronic Obstructive Pulmonary Disease; **CR**, Controlled Release; **DVA,** Department of Veterans’ Affairs; **GP**, General Practitioner; **IR**, Immediate Release; **LTCFs**, Long Term Care Facilities; **NH**, Nursing Homes; **PBS**, Pharmaceutical Benefits Scheme; **PRN**, Pro Re Nata; **RACFs**, Residential Aged Care Facilities; **RCT**, Randomised Controlled Trial; **RMMR**, Residential Medication Management Review; **SIMPLER**, SImplification of Medications Prescribed to Long-tErm care Residents.

**Table S2.** Specific types of opioids used in Australian ACFs.

| **Author, year** | **Opioid prevalence outcome measurements** | **Sample size** | **Overall opioid use n (%)** | **Data on specific opioids n (% of residents taking opioids out of entire study sample)** | | | | | | | | |
| --- | --- | --- | --- | --- | --- | --- | --- | --- | --- | --- | --- | --- |
|  |  |  |  | **Buprenorphine** | **Codeine** | **Fentanyl** | **Hydromorphone** | **Morphine** | **Oxycodone** | **Oxycodone + naloxone** | **Tapentadol** | **Tramadol** |
| **Administered** | | | | | | | | | | | | |
| Tan 2015^27a^  Tan 2016^8a^ Leung 2015^24a^ | Residents administered regular and/or PRN opioid(s) over 24-hours | 383 | 110 (29%) | 47 (12%) | Paracetamol/codeine  9 (2%)^b^ | 21 (6%) | - | 4 (1%) | 21 (6%) | 15 (4%) | - | 7 (2%) |
| Hussein 2019^30^ | Residents administered an opioid(s) over 24-hours | 458 | 95 (21%) | 17 (4% of total population, 18% of residents taking opioids) (Norspan®) | 4 (1% of total population, 4% of residents taking opioids) (Paracetamol with codeine CM strong pain relief® [1], Panadeine® [3]) | 4 (1% of total population, 4 % of residents taking opioids) (Durogesic® Patch) | 4 (1% of total population, 4% of residents taking opioids) (Dilaudid® [3], Jurnista® [1]) | 5 (1% of total population, 5% residents taking opioids)  (Oral liquid Ordine®) | 25 (6% of total population, 26% of residents taking opioids) (OxyContin®  [4], OxyNorm®  [3], Endone®  [18]) | 34 (7% of total population, 36% of residents taking opioids) (Targin®) | 13 (3% of total population, 14% of residents taking opioids) (Palexia®) | 13 (3% of total population, 14% of residents taking opioids) (Zydol® [7] and Zydol CR® [6]) |
| Picton 2021^40c^ | Residents administered PRN opioid(s) over 12-months* | 392 | 166 (42%) | - | Paracetamol/codeine  16 (4%) | - | - | Inj  89 (23%)  Oral  12 (3%) | - | - | - | - |
| **Prescribed** | | | | | | | | | | | | |
| Picton 2021^40c^ | Residents prescribed PRN opioid(s) as assessed over 24-hours (1^st^ July 2016)* | 392 | 118 (30%) | - | Paracetamol/codeine  20 (5%) | - | - | Inj  34 (9%)  Oral  4 (1%) | - | - | - | - |
| Stasinopolous 2018^26a^ | Residents prescribed PRN and regular + PRN oxycodone in the over 7 days* | 383 | N/A | - | - | - | - | - | PRN  72 (19%)  Reg+PRN  14 (19% of those with PRN charted, 4% total residents) | - | - | - |
| Veal 2014^28d^  Veal 2015^29d^ | Residents prescribed regular and/or PRN opioid(s) as per RMMR record over the study period | 7309 | Reg  2057 (28%)  PRN  782 (11%) | Patch  13% of total, 32% residents taking opioids | Codeine ± combination 8% of total, 20% of residents taking opioids ^e^ | Patch  7% of total, 18% of residents taking opioids | - | IR  4% of total, 11% of residents taking opioids  Inj  3% of total, 8% of residents taking opioids  CR  1% of total, 3% of residents taking opioids | IR  15% of total, 36% of residents taking opioids  CR  7% of total, 17% of residents taking opioids | - | - | 4% of study sample, 9% of residents taking opioids |
| Veal 2019^39^ | Residents prescribed regular and/or PRN opioid(s) as assessed on the audit date (24-hours)* | 477 | Reg  139 (29%)  PRN  169 (35%) | Patch (reg)  73 (15% of total, 53% of residents on regular opioids) | Tramadol or paracetamol + codeine (reg)  17 (4% of total, 12% of residents on regular opioids)  Codeine phosphate (PRN)  12 (3% of total, 7% of residents on PRN opioids)  Paracetamol + codeine (PRN) 36 (8% of total, 21% of residents on PRN opioids) | Patch (reg)  5 (1% of total, 4% of residents on regular opioids)  Inj (PRN)  1 (0.2% of total, 0.6% of residents on PRN opioids) | Reg  2 (0.4% of total, 1% of residents on regular opioids) | ER (reg)  5 (1% of total, 4% of residents on regular opioids)  Syringe driver (reg)  4 (1% of total, 3% of residents on reg opioids)  IR liquid (reg) 2 (0.4% total, 1% of residents on reg opioids)  Inj (PRN)  38 (8% of total, 23% of residents on PRN opioids)  Liquid (PRN): 19 (4% of total, 11% residents on PRN opioids) | IR (reg)  11 (2% of total, 8% of residents on regular opioids)  ER (reg)  8 (2% of total, 6% of residents on regular opioids)  IR (PRN)  68 (14% of total, 40% of residents on PRN opioids) | Reg  28 (6% of total, 20% of residents on regular opioids)  PRN  1 (0.2% of total, 0.6% of residents on PRN opioids) | Reg  1 (0.2% of total, 0.7% of residents on regular opioids) | Reg: see ‘codeine’  PRN  11 (2% of total, 7% of residents on PRN opioids) |
| Alderman 2018^43^ | Opioid(s) prescribed as per RMMR report over the study period | 22319 | N/A | 2139 (10%) | 1122 (5%) | 750 (3%) | 97 (0.4%) | 1061 (5%) | 5362 (24%) | - | 75 (0.3%) | 736 (3%) |
| **Dispensed** | | | | | | | | | | | | |
| Kalisch Ellett 2019^41^ | Veterans dispensed opioid(s) during the 1-year study period* | 14237 | 7049 (50%) | Patches  2676 (19%) | - | Patches  910 (6%) | - | 113 (1%) | 4031 (28%) | - | - | 714 (5%) |
| Roughead 2008^36^ | Veterans with at least one dispensing of the opioid(s) in the 3-month study period | 16126 | 3308 (21%)  *** | - | 1096 (7%) | - | - | - | 515 (3%) | - | - | 975 (6%) |
| Dolton 2012^42^ | Residents who received one or more dispensing’s of tramadol during the 2-year study period* | 3876 |  | - | - | - | - | - | - | - | - | 255 (7%) |
| Liu 2019^32i^ | Residents prescribed opioid(s) based on pharmacy records during the 1-year study period^g^ | 541 | - | 88 (16%) | 8 (2%)^h^ | 61 (11%) | - | - | 110 (20%) | - | - | - |

* as confirmed with author

+Methadone and pethidine not included in table as only reported for one study.

**a.** Data from the same study sample; **b.** Note: if codeine or dihydrocodeine per unit dose was <20mg, the product was classified under paracetamol; **c.** Reported point prevalence (prescribed) and period prevalence (administered) in the same study; **d.** Utilises data from the same study sample. Reports percentages in manuscript only. The cumulative percentages of those taking opioids equals more than 100% as the average number of opioids used per resident (using opioids) was 1.9; **e.** Opioids used predominantly as antitussives (dihydrocodeine or pholcodine), or codeine linctus with a direction indicating that its use was for cough or diarrhoea, were not included as analgesics; **f.** Paracetamol with codeine, tramadol. Oxycodone, other opioids (N02AA03, N02AA01, N02AB); **g.** Prevalence = number of residents prescribed the opioid during the 1-year period (regardless of frequency of use) as determined from the following sources: 1) pharmacy data records from nursing home contracted pharmacists; (2) facility-based medication charts when pharmacy data could not be obtained or (3) Pharmaceutical Benefits Scheme (PBS) data; **h.** As per ATC code (R05DA04) - codeine used as an analgesic (plain codeine product), or for other purposes (e.g., respiratory); **i.** Information obtained from supplementary file.

**Abbreviations; ER**, Extended Release; **INJ**, Injection; **IR**, Immediate release; **PRN**, Pro Re Nata; **Reg**, Regular; **RMMR**, Residential Medication Management Review; **SIMPLER**, SImplification of Medications Prescribed to Long-tErm care Residents

**Appendix 2. JBI Critical Appraisal Checklist for Prevalence Studies^21^**

*An adapted version of the checklist was used to appraise the quality of how opioid prevalence was assessed in all studies (i.e., not the overall quality of the study), irrespective of whether the study was opioid-related.*

*Criteria 1,2 and 3 refer to sampling methods. Criteria 4,5,6 and 7 refer to the quality of how opioid prevalence was assessed in each study. Each criterion was rated as a “Yes”, “No”, “Unclear” or “Not applicable (N/A)”. Where reporting was insufficient information to assess against a certain criterion, it was marked as unclear.*

| ***1. Were study participants sampled appropriately?***  *Authors should report how sampling was performed. Random probabilistic sampling from a defined subset of the population (sample frame) should be employed in most cases, however, random probabilistic sampling is not needed when everyone in the sampling frame will be included/analysed. Authors should provide clear resident inclusion and exclusion criteria, developed prior to recruitment. Was it clear that participants were residents of Australian RACFS? (i.e., Do the characteristics, demographics reported in the study represent residents of Australian RACFs?). Authors should sample participants from more than one facility.* | *☐ Yes*  *☐ No*  *☐ Unclear*  *☐ N/A* |
| --- | --- |
| ***2. Was the sample size adequate?***  *The larger the sample, the narrower the confidence interval around the prevalence estimate will be, making the results more precise. In cases of large sample sizes (e.g., large national studies), sample size can be considered adequate. If the authors did not conduct a sample size calculation, mark as “Unclear”.* | *☐ Yes*  *☐ No*  *☐ Unclear*  *☐ N/A* |
| ***3. Were the study subjects and the setting described in detail?***  *Authors should provide a comprehensive description of study sample (e.g., age, sex, diagnoses) AND description of the study nursing home setting (e.g., location, time-period of the study).* | *☐ Yes*  *☐ No*  *☐ Unclear*  *☐ N/A* |
| ***4. Was it specified whether regular or PRN data were collected?***  *Authors should specify whether regular or PRN opioids were collected and reported. If PRN data were not included, it should be stated in the exclusion criteria. For studies using pharmacy administrative data, it is not possible to discern between regular and PRN, mark as N/A* | *☐ Yes*  *☐ No*  *☐ Unclear*  *☐ N/A* |
| ***5. Was opioid prevalence measured in terms of administration to residents?***  *Did the authors measure opioid administration data? Opioid administration provides arguably the most accurate reflection of resident consumption.* | *☐ Yes*  *☐ No*  *☐ Unclear*  *☐ N/A* |
| ***6. Was the exposure (opioid use) measured in a standard, reliable way for all participants?***  *Authors should clearly describe how opioid use was measured (e.g., qualifications, training or experience of those collecting data) and description of how opioid use was found using other data sources (e.g., medical records). Was the same method of data collection used for all participants?* | *☐ Yes*  *☐ No*  *☐ Unclear*  *☐ N/A* |
| ***7. Was there appropriate statistical analysis?***  *Authors should clearly report the numerator and denominator. Ideally, percentages should be given with confidence intervals. The methods section should be detailed enough to identify the methods for reporting prevalence estimates.* | *☐ Yes*  *☐ No*  *☐ Unclear*  *☐ N/A* |

**Table S3.** Summary of quality assessment adapted from JBI Critical Appraisal Checklist for Prevalence Studies^21^

| **Author, year** | **1.**  **Were study participants sampled appropriately?** | **2.**  **Was the sample size adequate?** | **3.**  **Were the study subjects and the setting described in detail?** | **4.**  **Was it specified whether regular and/or PRN data were collected?** | **5.**  **Was opioid prevalence measured in terms of administration to residents?** | **6.**  **Was opioid prevalence measured in a standard, reliable way for all participants?** | **7.**  **Was there appropriate statistical analysis?** |
| --- | --- | --- | --- | --- | --- | --- | --- |
| Alderman, 2018^43^ | Yes | Yes | No | No | No | Unclear | Yes |
| Dolton, 2012^42^ | Yes | Unclear | Yes | N/A | No | Unclear | Unclear |
| Hussein, 2019^30^ | Yes | Unclear | Yes | Yes | Yes | Yes | Yes |
| Inacio, 2020^23^ | Yes | Yes | Yes | N/A | No | Yes | Yes |
| Jokanovic, 2017^31^ | Yes | Unclear | Yes | Yes | No | Yes | Yes |
| Kalisch Ellett, 2019^41^ | Yes | Yes | No | N/A | No | Unclear | Yes |
| Leung, 2015^24^ | Yes | Yes | Yes | Yes | Yes | Yes | Unclear |
| Liu, 2019^32^ | Yes | Unclear | Yes | N/A | No | Unclear | Yes |
| McClean, 2002^33^ | Unclear | Unclear | No | No | No | Unclear | Unclear |
| Picton, 2021^40^ | Yes | Unclear | Yes | Yes | Yes | Yes | Yes |
| Pont, 2018^34^ | Yes | Unclear | Yes | No | Yes | Unclear | Unclear |
| Pu, 2020^22a^ | Yes | Unclear | Yes | Yes | Yes | Unclear | Yes |
| Raban, 2020^35^ | Yes | Unclear | Yes | N/A | No | Yes | Yes |
| Roughead, 2008^36^ | Yes | Yes | Yes | N/A | No | Unclear | Yes |
| Sharma, 2021^25^ | Yes | Yes | Yes | Yes | Yes | Yes | Yes |
| Snowdon, 2006^37^ | Yes | Unclear | Yes | Yes | Yes | Yes | Unclear |
| Stasinopoulos, 2018^26^ | Yes | Yes | Yes | Yes | No | Yes | Yes |
| Tan, 2015^27^ | Yes | Yes | Yes | Yes | Yes | Yes | Yes |
| Tan, 2016^8^ | Yes | Yes | Yes | Yes | Yes | Yes | Yes |
| Taxis, 2017^38^ | Yes | Unclear | No | N/A | No | Unclear | Unclear |
| Veal, 2014^28^ | Yes | Yes | Yes | Yes | No | Yes | Unclear |
| Veal, 2015^29^ | Yes | Yes | Yes | Yes | No | Yes | Unclear |
| Veal, 2019^39^ | Yes | Unclear | Yes | Yes | No | Yes | Yes |
